# Lingual Epithelial Stem Cells and Organoid Culture of Them

**DOI:** 10.3390/ijms17020168

**Published:** 2016-01-28

**Authors:** Hiroko Hisha, Toshihiro Tanaka, Hiroo Ueno

**Affiliations:** 1 Department of Stem Cell Pathology, Kansai Medical University, 5-1, 2-chome, Shin-machi, Hirakata-shi, Osaka 573-1010, Japan; hishah@hirakata.kmu.ac.jp (H.H.); tanakato@hirakata.kmu.ac.jp (T.T.); 2 Third Department of Internal Medicine, Kansai Medical University, 5-1, 2-chome, Shin-machi, Hirakata-shi, Osaka 573-1010, Japan

**Keywords:** lingual epithelium, stem cells, Bmi1, organoid

## Abstract

As tongue cancer is one of the major malignant cancers in the world, understanding the mechanism of maintenance of lingual epithelial tissue, which is known to be the origin of tongue cancer, is unquestionably important. However, the actual stem cells that are responsible for the long-term maintenance of the lingual epithelium have not been identified. Moreover, a simple and convenient culture method for lingual epithelial stem cells has not yet been established. Recently, we have shown that Bmi1-positive cells, residing at the second or third layer of the epithelial cell layer at the base of the interpapillary pit (IPP), were slow-cycling and could supply keratinized epithelial cells for over one year, indicating that Bmi1-positive cells are long-term lingual epithelial stem cells. In addition, we have developed a novel lingual epithelium organoid culture system using a three-dimensional matrix and growth factors. Here, we discuss current progress in the identification of lingual stem cells and future applications of the lingual culture system for studying the regulatory mechanisms of the lingual epithelium and for regenerative medicine.

## 1. Introduction

The mammalian tongue is an important digestive and sensory organ that has multiple functions such as food intake, sensing taste and touch, and serving as an articulatory organ. The surface of the tongue is covered with stratified squamous epithelial cell layers. Lingual dorsal epithelium contains four kinds of papillae: filiform, fungiform, foliate, and circumvallate papillae. Stratum corneum is seen in filiform papillae, but not in fungiform, foliate, or circumvallate papillae. In contrast, taste buds are seen in fungiform, foliate, and circumvallate papillae but not in filiform papillae. Lingual epithelium is replaced continually throughout the life of mammals, and the turnover of mouse lingual epithelium, at a rate of 6–7 days, is 4–5-fold higher than that of dorsal skin [1], suggesting the existence of stem cells in the papillae. A label-retaining assay using ^3^H-TdR revealed that lingual epithelial stem cells (LESCs) are located in the basal layer of the lingual epithelium as in other epithelial tissues [2]. Several research groups have proposed candidates for LESCs and progenitors. However, the stem cell markers used in these studies, keratin 5 and keratin 14, are not specific to stem cells since they are also detected in some of the mature epithelial cells. Therefore, the actual stem cells that are responsible for the long-term maintenance of the lingual epithelium have not been identified. In recent years, a powerful technique, *in vivo* lineage tracing, has been applied for the identification of LESCs. In this review, we introduce and discuss current progress in the identification of LESCs.

To identify tissue-specific stem cells in the adult, a primary culture system that can reproduce the physiological environment *in vitro* and allow the differentiation of stem cells into various kinds of mature cells needs to be established. Using this system, we can precisely examine the pluripotency and the growth factor requirements of the stem cells. Recently, a three-dimensional (3D) organoid culture technique using extracellular matrix has been developed for the small intestine [3], stomach [4], and colon [5]. This technique allows the generation of organoids containing multilayered epithelial structures from crypts and even from single stem cells isolated from adult animals. In this review, we introduce a new lingual epithelial organoid culture system as well as early lingual epithelial cell culture systems.

## 2. Lingual Stem Cell Markers

### 2.1. Keratin 5 and Keratin 14

Keratin 5 (K5) and keratin 14 (K14), intermediate filament proteins, are known to be expressed in basal keratinocytes of stratified epithelium in the skin, and the mutation or absence of both proteins makes the cellular architecture in basal keratinocytes vulnerable [6]. Similar to the skin, immunohistochemistry analyses of mouse tongue revealed that both proteins are expressed at the highest level in the basal layer of the tongue epithelium. The expression level decreases in each layer closer to the surface epithelial layer [7,8] (Table 1). Luo *et al.* reported that K5-positive lingual epithelial cells (LECs) obtained from K5-eGFP mice could generate a multilayered squamous keratinized epithelium when these cells were cultured on a collagen-fibroblastic cell-matrix in the presence of epidermal growth factor (EGF) and fibroblast growth factor 7 (FGF7) [9], supporting that K5-positive cells include lingual stem cells and/or progenitors.

**Table 1:** Markers of lingual epithelial stem cells (LESCs) and the results of their lineage tracing experiments.

Marker	Keratin 14	NTPDase2	Bmi1	Tcf3
Position of Maker-Positive Cells in Lingual Epithelium	Basal Layer and Mature Keratinized Cell Layer	Basal Layer, Suprabasal Layer and Taste Bud	The 2nd or 3rd Layer from Basal Layer (1 Cell per IPP)	Basal Layer (2-5 Cells per IPP)
Differentiation to Papillae	Yes (Filiform and Fungiform Papillae)	Yes (Filiform, Fungiform and Circumvallate Papillae)	Yes (Filiform Papillae Only)	Yes (Filiform Papillae Only)
Differentiation to Taste Buds	Yes	Yes	No	(Not Described)
Period of Chase	30 days	150 days	336 days	180 days
Reference	[7]	[10]	[8]	[11]

IPP: interpapillary pit.

Using an *in vivo* lineage tracing assay with *K14-Cre ER:Rosa26-LacZ* mice, Okubo *et al*. demonstrated that K14-positive cells give rise to mature keratinocytes in both filiform and fungiform papillae and in taste bud cells [7]. However, K14 is detected in not only Ki67-positive proliferating cells but also many of the mature keratinized epithelial cells in the interpapillary pit (IPP). Moreover, in the study, the fate of the K14-positive cell-derived cells were followed for only 30 days; therefore, it is not clear whether K14-positive cells are actual long-term stem cells. Based on both the expression patterns (immunostaining results) of several proteins (K5, K14, Sox2 [SRY {Sex Determining Region Y}-Box 2], and Trp63 [p63, a member of the p53 transcription factor family]) in the lingual epithelium and taste buds and the K-14 lineage tracing analyses, they proposed a model in which K5-, K14-, and Trp63-positive, but Sox2-low-positive basal cells are long-term progenitor cells that generate keratinocyte lineage cells. Moreover, they considered that K5-, K14-, Trp63-, and Sox2-positive cells adjacent to taste buds can give rise to both keratinocytes and taste bud cells.

### 2.2. NTPDase2

Ecto-nucleoside triphosphate diphosphohydrolase 2 (NTPDase2) is a plasma membrane nucleoside triphosphate that has the ability to hydrolyze extracellular nucleoside triphosphates to diphosphates. The enzyme is expressed on neural stem cells of the subventricular zone [12]. Bartel *et al.* found that NTPDase2 colocalized with the glial glutamate/aspartate transporter (GLAST), which is regarded as a marker of type I cells in taste buds, by using immunohistochemical and enzyme histochemical staining methods [13]. In contrast, Li *et al.* demonstrated that LECs in basal and suprabasal cell layers as well as taste bud cells in fungiform and circumvallate papillae express NTPDase2 by using *in situ* hybridization with an NTPDase2 probe [14] (Table 1). Moreover, a genetic tracing study of NTPDase2-positive cells (doxycycline inducible, NTPDase2-rtTA/TeTO-Cre; RosaLacZ reporter system) revealed that descendant cells derived from the NTPDase2-positive cells generated filiform, fungiform, and circumvallate papillae as well as taste bud cells in fungiform papillae and circumvallate papillae. From the results, they propose the existence of common progenitor cells that contribute to both taste bud cells and LECs. However, by the single-color lineage tracing method using the Rosa26 reporter mouse in this study, the proof for the bipotency of K14 positive lingual stem/progenitor cells was not sufficient, because the different clones next to each other could show the same color.

### 2.3. Multicolor Lineage Tracing Method

**Figure 1:**
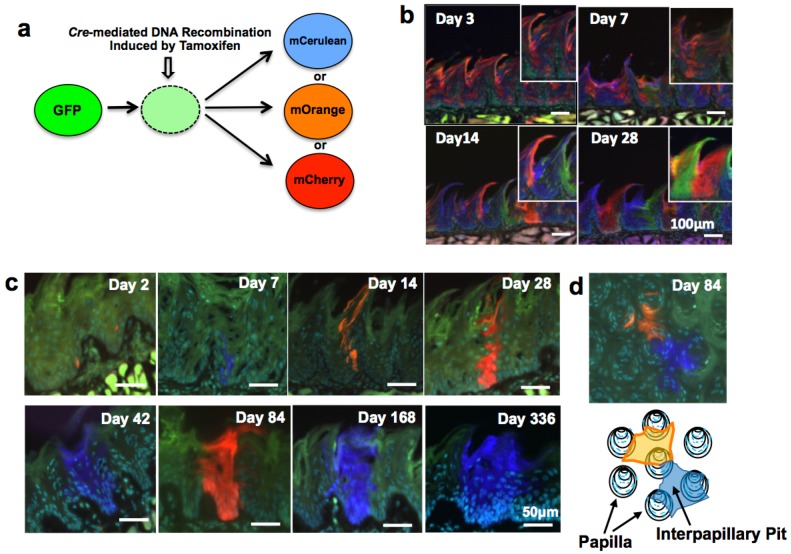
(**a**) *Cre*-mediated fluorescent color change. Fluorescent labels of cells change from green to either red, orange, or blue upon tamoxifen-induced *Cre*-mediated excision of floxed cassettes; their descendant cells retain the new color; (**b**) Individual cells of *Rosa26CreERT2; Rosa26-rainbow* mice injected with tamoxifen were labeled and analyzed at the indicated time points. Magnified images are shown at the right upper corner of the images; (**c**) Fate of cells derived from Bmi1-positive cells. *Bmi1-CreER*; *Rosa26-rainbow* mice were injected with tamoxifen and analyzed at the indicated time points; (**d**) Areas maintained by single Bmi1-positive stem cells. Three (and less frequently four) filiform papillae displayed different colors, suggesting that Bmi1-positive LESCs are present in the middle of the pit. This figure is a modified version of figures presented in our previous report [8].

To precisely examine the fate of each stem cell, the multicolor lineage tracing method is now considered as one of the most powerful techniques. The multicolor lineage tracing method was originally developed to analyze lineage relationships between blood and endothelial cells within yolk sac blood islands of mice [15]. However, in the original method, multicolor chimeras were generated by injecting multiple kinds of colored ES cells into blastocysts, which were then transplanted back to the uterus of pseudopregnant mice. By this method, the timing of the generation of multicolor chimeras was fixed at an early stage of development; therefore, application of the method to study adult tissue-specific stem cells was limited. To overcome the problem, we developed a mouse model for inducible multicolor lineage tracing, *Rosa26-rainbow* mice. In the *Rosa26-rainbow* mice, *Cre*-mediated excision of floxed cassettes is induced by tamoxifen in all of the cells in the body (including tissue-specific stem cells), causing the fluorescent label of every cell to change from green to one of three colors (red, orange, or blue) (Figure 1a) [16,17]. The descendant cells of the red, orange, or blue stem cells retain the color of the parent stem cells, and, therefore, this system is convenient to examine the fate and dynamics of individual normal and cancer stem cells [18]. We used this system to follow the fate of individual LESCs *in vivo* and found that clonal expansion of single-color cells occurred in each IPP. In addition, each mature pit contained a single-color cell cluster of LECs (red, orange, or blue) (Figure 1b) [8]. This finding indicates that single LESCs in each IPP have the ability to generate LECs in the whole pit by 28 days after tamoxifen administration.

### 2.4. Bmi1

Bmi1 belongs to the polycomb group family and has an important role in cell cycle, self-renewal, and maintenance of hemopoietic and neural stem cells [19,20]. *In situ* hybridization for *Bmi1* (B cell-specific Moloney murine leukemia virus integration site 1) showed that Bmi1-positive cells reside in the second or the third layer above the basal layer of mouse epithelium; we never observed more than two Bmi1-positive cells in one IPP [8] (Table 1). From multicolor lineage tracing using tamoxifen-inducible *Bmi1-CreER*; *Rosa26-rainbow* mice, it was shown that one labeled cell was detected per IPP two days after tamoxifen injection. The descendants of Bmi1-positive cells expanded and occupied the entire IPP by four to 12 weeks after tamoxifen induction (Figure 1c). Finally, the descendant cells generated filiform papillae, and the single-color area generated by single stem cells was maintained for at least 336 days (Figure 1c), indicating that Bmi1-positive cells in the IPP are long-term LESCs. It was also shown that a single Bmi1-positive stem cell supplied the LECs for three (or less frequently four) neighboring filiform papillae (Figure 1d).

Using the lineage tracing experiments, we have shown that Bmi1-positive cells usually were resting or slow-growing. However, after irradiation-induced injury, the cells rapidly entered the cell cycle and regenerated tongue epithelium. These results suggest that Bmi1-positive LESCs are important for the tissue maintenance of lingual epithelium in the physiological state as well as for rapid regeneration after injury.

Immunohistochemistry staining of human normal oral mucosal tissues showed that Bmi1-positive cells were weakly detected in the basal layer of epithelium, but not in the upper layers [21]. Bmi1 expression was also observed in the basal layer of the lingual epithelium [22]. These results suggest the possibility that Bmi1 is a marker of stem cells in human oral mucosa and lingual epithelium as well as in mouse lingual epithelium.

Bmi1 is now considered to be a ubiquitous stem cell biomarker expressed on various stem cells such as hemopoietic [19], neural [20] and intestinal [23] stem cells. Our more recent study using the multicolor lineage tracing technique revealed that Bmi1-positive cells residing in the inner periphery of seminiferous tubules were long-term germ stem cells that could produce sperm for at least 48 weeks [24], and that Bmi1-positive cells residing in sweat glands of the acral epithelium (does not contain hair follicles or sebaceous glands, such as on the palms and soles) were reserver stem cells that were slow-cycling but occasionally entered the cell cycle and then generated the acral epithelium [25].

### 2.5. Tcf3

Tcf3 (a member of the Lef/Tcf family) is a transcription factor important for regulating embryonic cell function and adult normal/malignant stem cell function [26]. It is also expressed in hair follicle bulges (stem cell niche in the skin) [25] (Table 1). Howard *et al.* recently demonstrated that Tcf3 is expressed on overall cells of the basal layer and in some cells of the suprabasal layer in the mouse lingual epithelium [11]. Lineage tracing experiments using *Tcf-GC*; *Rosa26-mTmG* mice detected 2–4 labeled cells in the basal layer of the IPP 3 days after tamoxifen administration; the basal clones continued to expand, finally occupying the entire interpapillary epithelium and filiform papillae. After the chase of six months, the neighboring 3–4 filiform papillae were labeled green, indicating that the Tcf3-positive clones supplied epithelial cells for 3–4 filiform papillae. This result is consistent with our previous observation (Figure 1d) [8]. Immunostaining for Tcf3 protein revealed that the majority of Tcf3-positive cells in the basal layer were Bmi1-negative, but one or two Tcf3- and Bmi1-double positive cells were detected per IPP. Bmi1-positive/Tcf3-negative cells were observed, leading Howard *et al.* to conclude that the expression of these proteins is not directly linked. They further demonstrated that there is no direct relationship between Tcf3 expression and proliferation status (examined by immunostaining of Ki67). Overall, it was suggested that Tcf3 expression is not restricted to actual LESCs but is expressed in more differentiated LECs (LESCs, transit-amplifying cells, and keratinized epithelial cells).

## 3. Culture of Lingual Epithelial Cells

### 3.1. Organ Culture

According to previous studies on tongue development, the tongue epithelium remains histologically immature until the 13th day of gestation, and pre-papilla placodes and fungiform papillae appear on the 14th day and 15th day, respectively. Organ culture of rat embryonic tongues was performed to examine the morphogenesis of lingual epithelium. Entire tongues were taken from rat embryos on the 13th day of gestation and incubated at the air/liquid interface in the presence of medium containing fetal bovine serum (FBS) and B-27. After two days of culture, fungiform papillae were observed in the epithelium, a stage of development equivalent to the tongue of an intact embryo on the 15th day of gestation [27] (Table 2). In the same laboratory, it was shown that the addition of noggin to the organ culture system increased the number of fungiform papillae, whereas bone morphogenic protein (BMP) 2, 4, or 7 inhibited papilla formation, suggesting that noggin and BMPs, both localized within papilla placodes, have opposite effects on the morphogenesis of lingual epithelium [28] (Table 2).

A rolling organ culture system also has been used for mouse embryonic tongues on day 12.5 or 13.5 of gestation [29,30] (Table 2). In this system, the tongues were cultured in medium containing various cytokines, antibodies, or enzyme inhibitors; thus, the effects of the molecules on the developing tongues could be precisely examined.

### 3.2. Culture on Extracellular Matrix

The application of the organ culture system for adult tongue is difficult because the proliferation and differentiation potential of adult tissues is much lower than embryonic tissues. Lingual epithelial cell culture of adult LECs on extracellular matrix was reported by Ookura *et al.* They obtained integrin β1 (a keratinocyte stem cell marker)-positive LECs from adult mouse tongues by using a magnetic cell separation system and cultured them on a collagen gel/Matrigel-coated dish in the presence of EGF and basic fibroblast growth factor 2 (FGF-2) [31] (Table 2). The integrin β1-positive LECs proliferated on the extracellular matrix and generated a monolayer with epithelial morphology. However, a stratified keratinized epithelial layer was not generated in this culture system.

**Table 2:** List of lingual epithelial cell (LEC) cultures.

Culture System	Culture Setup (Day 0) Imaged		Advantage	Limitation	Ref.
**Organ Culture**	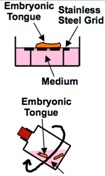		Close to physiological conditions	Impossible to apply to adult tongues Difficult to maintain long-term growth (maximum 6 days)	[27,28,29,30]
**Culture on Extracellular Matrix**	2D	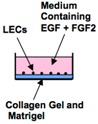	Possible to generate epithelial cell monolayer from single cells	Impossible to generate stratified keratinized epithelial cell layer	[31]
**Organotypic Raft Culture**	3D	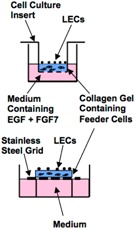	Possible to generate stratified keratinized epithelial cell layer from single cells Also possible to observe inversion activity and its process into collagen gel layer of malignant LECs	Prior preparation of the feeder layer is required Results of culture experiments are generally influenced by feeder cells	[9,32,33]
**Organoid Culture**	3D	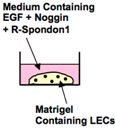	Simple and easy in culture technique Possible to generate stratified keratinized epithelial cell layer from single cells Also possible to trace the fate of each LEC	High concentration of growth factors is required	[34]

### 3.3. Organotypic Raft Culture

To achieve a more suitable environment for the proliferation and maturation of LECs, an organotypic raft culture system was developed by Luo *et al.* [9] (Table 2). K5-positive cells, obtained by cell sorting LECs from K5-eGFP mice, were placed on the top of collagen gel containing 3T3 feeder cells. The collagen gel in a cell culture insert was cultured at the air-liquid interface in the presence of EGF and FGF7. This procedure generated a multilayered squamous keratinized epithelium expressing high levels of K14 and Trp63 [9]. The limitations of this system include the requirement for a specific cell separation procedure (namely K5-positive cell separation) to induce sufficient epithelial cell growth and the low colony-forming efficiency (only 0.78%) of the K5-positive cells [9].

The organotypic raft culture system has been applied in studies on carcinoma cell invasion. Nurmenniemi *et al.* cultured an invasive human tongue squamous cell carcinoma cell line (HSC-3) on collagen gel containing gingival fibroblasts, where the gel was mechanically supported on a stainless-steel grid [32] (Table 2). They measured the invasion depth and area of HSC-3 cells into the collagen gel layer, thereby assessing the invasion activity and the process used by HSC-3 cells. It is known that the cancer environment is mainly composed of cancer-associated fibroblasts. However, other cells, such as endothelial cells and inflammatory cells, also affect cancer progression, invasion, and recurrence. Therefore, Nurmenniemi *et al.* used real human tissue, uterine leiomyoma, instead of collagen gel containing gingival fibroblasts and observed a much higher invasion activity for HSC-3 cells into the myoma tissues. Similar results were obtained by Bitu *et al.* [33] (Table 2), and, therefore, the myoma organotypic raft culture method is considered advantageous for studies on tongue squamous cell carcinoma.

### 3.4. Organoid Culture

**Figure 2:**
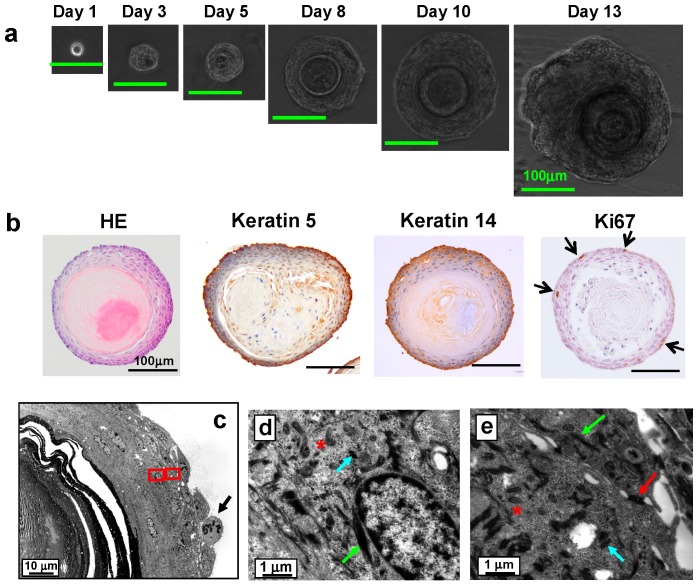
(**a**) Time course of lingual organoid growth. LECs were cultured in the organoid culture system, generating round-shape organoids with a concentric cell arrangement. Phase-contrast image; (**b**) Histological analysis of organoids. Organoids were separated from Matrigel and their paraffin sections were stained with HE reagents and anti-K5 and K14 antibodies. K5 and K14-positive cells were observed in the outer periphery of the organoids. Staining for Ki67 showed that some cells actively proliferated in the outer periphery (arrows); (**c**) Transmission electron micrograph of organoids. The organoids had a stratum corneum in the center and flattened cells surrounding the stratum corneum. At the periphery of the organoid, a cell with aggregated chromosomes (indicated by arrow) was detected; (**d**,**e**) Higher magnification views of the boxed areas in (**c**). Many keratohyaline granules (blue arrow), keratin fibers (green arrow), and lamellar granules (indicated by asterisk) were detected in the cytoplasm. Moreover, formation of desmosomes between cells was observed (red arrow). Modified from [34].

To generate a multilayered keratinized epithelium at a high efficiency from unseparated (whole) lingual epithelial cell populations, we attempted a three-dimensional (3D) lingual epithelial cell culture system [34] (Table 2). Enzymatically isolated LECs were cultured in Matrigel in the presence of EGF, noggin, and R-spondin1. Round-shaped organoids with concentric cell arrangements were generated at a seeding efficiency of 3.4% (Figure 2a). The organoids showed a stratum corneum (stained red with eosin; Figure 2b). Positive staining for anti-K5 and K14-antibodies was observed in the outer periphery of the organoids, indicating the localization of LESCs and progenitor cells. We deduce that differentiated cells migrate toward the center of organoids according to their maturation. Several Ki67-positive cells (proliferating cells) were observed in the outer periphery of the organoids (indicated by arrows), and we speculate that cell mitosis occurred mainly at these sites (Figure 2b). A transmission electron micrograph of the organoid confirmed the existence of a stratum corneum in the center with flattened cells surrounding the stratum corneum (Figure 2c). On the outer side of the organoid, a cell with aggregated chromosomes (indicated by black arrow) was observed, suggesting that mitosis had been actively occurring at this site. Figure 2d,e is higher magnification photographs of the areas enclosed in the squares in Figure 2c, and granules and fibers specific to cells in the intermediate epithelium are shown in both figures. In addition, the presence of desmosomes between the LECs was observed (red arrow; Figure 2e).

**Figure 3:**
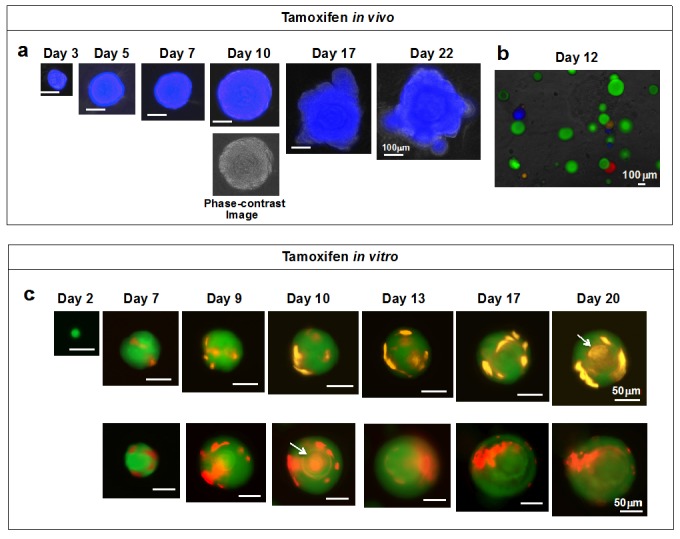
Potential of Bmi1-positive cells to generate organoids. (**a**) Time course of organoid growth (*in vivo* administration of tamoxifen). Two days after administering tamoxifen, LECs from *Bmi1-CreER*; *Rosa26-rainbow* mice were cultured in the organoid culture system for three days. Induction of *Cre* labeled individual cells red, orange, or blue, and the organoids showed mosaic patterns composed of the colored cells on day 5 of culture. Fluorescence image; (**b**) Representative photographs of organoids on day 12 of culture (*in vivo* administration of tamoxifen). Most organoids were green, but some round-shape organoids were red, orange, or blue. Overlay of fluorescence image and phase-contrast image; (**c**) Time course of organoid growth (*in vitro* administration of tamoxifen). Active form of tamoxifen was added to the LECs from *Bmi1-CreER*; *Rosa26-rainbow* mice at the start of culture. Bmi1-positive cells were induced by the tamoxifen to turn red or orange and formed colored stratum corneum (indicated by arrows). Modified from [34].

To examine whether Bmi1-positive cells have the potential to generate organoids, we injected *Bmi1-CreER*; *Rosa26-rainbow* mice with tamoxifen. Two days later, the LECs were collected and cultured in our organoid culture system. Figure 3a shows the time course of the formation of a representative blue-colored organoid. A phase-contrast image at day 10 shows that the organoid has a concentric cell arrangement. As shown in Figure 3b, all organoids were of a single color, and organoids containing more than one color were never observed. From this result, it is evident that each colored organoid was generated from a single Bmi1-positive cell. The findings clearly show that single Bmi1-positive cells have the potential to generate an organoid.

Next, tamoxifen was added to the *Bmi1-CreER*; *Rosa26-rainbow* mouse organoid culture system at the beginning of culture to induce *Cre*-mediated recombination in the Bmi1-positive cells. On day 7 of the culture, a few orange or red cells were detected at the outer periphery of the green-colored organoids (Figure 3c). Thereafter, the orange and red cells proliferated rapidly, some spreading toward the center of the organoids to form a stratum corneum (indicated by arrows). All of the organoids were green with either orange or red cells. This indicates that only one Bmi1-positive cell existed per organoid at the time of tamoxifen administration.

These findings (Figure 3) have confirmed our previous study [8] showing that Bmi1-positive cells are LESCs. The above studies demonstrate that each organoid is generated from a single cell and that single LESCs can differentiate into mature keratinized LECs. Therefore, our organoid culture system is useful for identifying LESCs.

## 4. Consideration of the Lingual Stem Cell Niche

The stem cell niche for lingual epithelial stem cells is unknown. Here, we discuss candidate niche cells deduced from other stem cell systems. In the intestine, Paneth cells and myoepithelial cells located at crypt bottoms have been reported to function as niche cells of Lgr5-positive intestinal stem cells [35]. Notably, Paneth cells are terminally differentiated cells generated by intestinal stem cells, indicating that intestinal stem cells are at least partially supported by their own descendant cells. On the other hand, other niche cells located at the crypt bottom, defined as myoepithelial cells of mesenchymal origin, do not originate from intestinal stem cells.

In the tongue, we reported that Bmi1-positive cells were not located at the base of the epithelial layers; instead, these cells were present most frequently in the second or third layer [8]. Therefore, we speculate that surrounding cells generated by lingual epithelial stem cells could be candidate niche cells.

In the bone marrow, several concepts for the hematopoietic stem cell niche were proposed in the 2000s; these include the osteoblastic niche [36], reticular cell niche [37], and perivascular niche [38]. Recently, the arteriolar niche [39] and perisinusoidal niche [40] were also proposed. Yamazaki *et al.* demonstrated that Schwann cells were components of the perivascular niche and had the ability to induce a dormancy state in hematopoietic stem cells [41].

**Figure 4:**
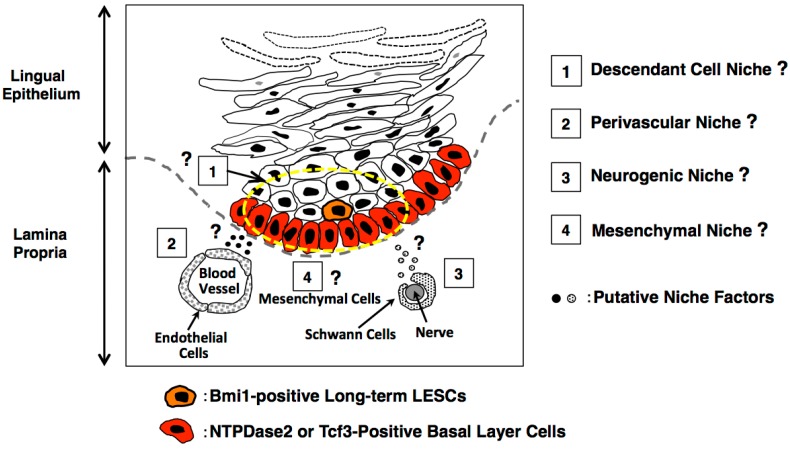
Putative lingual stem cell niche deduced from other stem cell systems.

Based on the concepts of the stem cell niche proposed in various tissues, it is possible that similar niche systems, such as the descendant cell niche, perivasucular niche, mesenchymal niche, and neurogenic niche, also function in the lingual epithelium (Figure 4). Further characterization of the lingual epithelium is needed to uncover niche cells controlling actual LESCs and to determine the physiological roles of niche cells for the maintenance and regeneration of the epithelium. Our lingual organoid culture system [34] is extremely useful for the screening of niche factors and the identification of niche cells by coculture with isolated LESCs.

## 5. Applications and Perspectives

Tongue squamous cell carcinoma (TSCC) is known to occur with a high incidence relative to other types of oral cancer, and long-term physical and chemical stimulation is one of the causes of TSCC. Most TSCC is derived from LESCs that have acquired malignant changes. However, the mechanisms and causes of malignant changes in LESCs have not been elucidated. To determine the causes of cancer outbreak, identification of LESCs and their niche is necessary. We have shown that Bmi1-positive cells are long-term LESCs [8]. In TSCC, immunohistochemistry analyses revealed that the number of Bmi1-positive cells increased significantly according to malignant progression. Bmi1 expression levels were found to be correlated with advanced malignant features on TSCC, such as invasion, metastasis, chemoresistance, and poor prognosis [20,42]. In contrast, Hayry et al. demonstrated that negative Bmi1 expression in TSCC predicted poor prognosis [43]. Bmi1 is a direct target of c-myc, and the c-myc-Bmi1-manganese superoxide dismutase (SOD2) pathway is related to the invasion and metastasis of TSCC [20]. Upregulation of Bmi1 expression induces enhanced chemoresistance to cisplatin in TSCC cells [42]. Thus, many studies have demonstrated that Bmi1 is a key molecule in the initiation or progression of TSCC. It is not clear whether the aberrant overexpression of Bmi1 was caused by malignant conversion of normal Bmi1-positive cells or whether Bmi1-negative cells acquired Bmi1 overexpression through unknown mechanisms before transforming into malignant cells. From the above-mentioned studies, it is clear that the Bmi1 molecule has a role in TSCC; however, further studies are needed to determine the details of this role.

Rapid and complete tongue regeneration should be induced after lingual tumor excision. For this purpose, polyglycolic acid sheets and fibrin glue sprays have been used for covering open wounds after lingual tumor excision [44]. Using our organoid culture system, we observed that immature organoids harvested on day 3 of culture, which did not have stratum corneum, could be engrafted and matured on the tongues of recipient mice [34]. Technical difficulties prevented injection of the organoids into the subepithelial area; thus, the grafted organoids expanded in the muscle layer rather than the epithelial layer of the recipient tongues. When techniques to inject the organoids directly into the subepithelium area can be developed, our culture system will be useful in lingual regeneration medicine to treat injury and for tumor excision.

We also observed that organoids generated from carcinogen (4-nitroquinolin 1-oxide)-treated mice had an abnormal morphology and a high number of Ki67-positive cells [34]. These results showed that the organoid culture system is useful for studying the regulatory mechanisms of normal lingual epithelium and the mechanism of carcinogenesis.
